# An open-label, proof-of-mechanism trial evaluating a neuroactive steroid GABA modulator in tinnitus

**DOI:** 10.3389/fneur.2025.1662226

**Published:** 2025-11-18

**Authors:** Luke S. Watson, Patricio O’Donnell, Kemi Bankole, Youssef Toubouti, Richard S. Tyler, Jason K. Johannesen

**Affiliations:** 1 Sage Therapeutics, Cambridge, MA, United States; 2 Department of Otolaryngology, Head and Neck Surgery, University of Iowa, Iowa, IA, United States; 3 Department of Communication Sciences and Disorders, University of Iowa, Iowa, IA, United States

**Keywords:** brexanolone, GABAergic inhibition, neurotology, otolaryngology, excitatory-inhibitory balance

## Abstract

Imbalances of excitatory and inhibitory signaling within the central auditory pathways are hypothesized to underlie tinnitus pathophysiology. Neuroactive steroids, such as allopregnanolone, are positive allosteric modulators of GABA_A_ receptors that regulate neuronal signaling in part through their binding affinity for and inhibitory action at extrasynaptic GABA_A_ receptors. This pilot, single-arm, proof-of-mechanism trial investigated whether brexanolone, an intravenous formulation of allopregnanolone, may improve tinnitus symptomatology. Adults between 18 and 65 years of age with bilateral, chronic, moderate tinnitus were recruited in this single-arm, open-label study. Ten participants received a 6-h infusion of brexanolone (30 min at 30 mcg/kg/h, 30 min at 60 mcg/kg/h, and 300 min at 90 mcg/kg/h). Primary outcomes were safety and tolerability, including treatment-emergent adverse events. Secondary outcomes were changes from baseline in visual analogue scale scores of tinnitus loudness and annoyance, assessed on day 1 as well as between days 2 and 7. All enrolled participants completed dosing day evaluations, and nine of them completed the final follow-up assessment on day 7. A single, unrelated, non-severe adverse event was reported. A clinically meaningful reduction in perceived tinnitus severity was detected as early as 2 h into the infusion period and remained throughout the post-dose observation week. Brexanolone was well tolerated and appeared to reduce the severity of experienced tinnitus and its related functional consequences relative to baseline. These results provide an important incremental advancement in support of a GABAergic hypothesis of tinnitus manifestation. Further evaluation in randomized placebo-controlled trials is needed to confirm these findings.

**Clinical trial registration:**

## Introduction

Tinnitus is the perception of sound (e.g., ringing, buzzing, humming, or clicking) in the absence of a corresponding sound source and is estimated to occur in approximately 1 in 10 adults in the US population (1). Most cases of tinnitus are classified as “subjective” in nature, wherein the tinnitus phenomenon is heard only by the affected individual. In extremely rare cases, termed “objective” tinnitus, the sound is not only heard by the affected individual but can also become audible to an examiner using a stethoscope or other recording device. Tinnitus can result from damage to the auditory system due to various causes, such as sensorineural hearing loss, Ménière’s disease, and chronic noise exposure (2, 3), with other health conditions such as dyslipidemia and insulin resistance more recently gaining recognition as additional risk factors (4, 5). Tinnitus is also commonly classified according to the duration of symptoms and is considered “acute” if experienced for less than 3 months, “sub-acute” if still experienced for more than 3 months, and “chronic” if experienced for periods of 6 months or longer (6). With greater severity, tinnitus can affect quality of life (7), with “bothersome” tinnitus referring to cases in which the characteristic sound becomes very disturbing and may be accompanied by symptoms of anxiety, depression, sleep disturbances, and maladaptive psychological reactions that impede daily function (6).

There are currently no Food and Drug Administration (FDA)-approved pharmacological treatments for tinnitus; however, the *γ*-aminobutyric acid (GABA) class of drugs, such as alprazolam (8) and clonazepam (9), and the GABA homolog gabapentin (10–12) have been investigated in clinical trials. GABA is an amino acid that functions as an inhibitory neurotransmitter for the central nervous system (13). Disruption in GABAergic signaling affects the balance between excitatory/inhibitory inputs to central auditory pathways. The GABA_A_ receptor is expressed throughout the auditory system, including the auditory nuclei, thalamus, and auditory cortex, and is highly concentrated in the cochlea, where specific receptor subunits associate with long-term maintenance of hair cells and inner ear neurons (14). Reductions in auditory cortex GABA levels occur with older age and correspond to elevated pure-tone hearing thresholds in humans (15). The inhibitory function of the GABA_A_ receptor is also critical for auditory processing, such as sound segmentation and multi-sensory integration, through the regulation of input/output functions, frequency tuning, and temporal response accuracy (16).

Animal models provide evidence linking tinnitus to GABAergic dysfunction (17) and are further supported by clinical observations. A significant decrease in GABA_A_ receptor density of the medial temporal cortex has been observed in patients with severe tinnitus using single-photon emission computed tomography (18). Additionally, GABA concentrations are also reportedly lowered in the auditory cortices of patients with tinnitus (19).

Despite multiple lines of evidence linking tinnitus to reductions in GABAergic inhibition, clinical trials examining the efficacy of drugs that act to increase GABA signaling provide only modest support (20). Considering these results, it is important to note that traditional approaches use GABAergic drugs, such as benzodiazepines, which bind primarily at synaptic GABA_A_ receptors and regulate receptor activation in a “phasic” input-dependent manner (21). In contrast, drugs that potentiate extrasynaptic GABA_A_ receptors exert “tonic” inhibition of network activity, arguably the more effective mechanism for alleviating excess excitatory activity implicated in tinnitus pathophysiology. For instance, gaboxadol, which selectively activates extrasynaptic GABA_A_ receptors, has demonstrated efficacy in animal models [reviewed in Richardson et al. (22)] likely by reducing spontaneous and evoked firing rates of sensory thalamic neurons and thus dampening hyperexcitability to the auditory cortex (23).

Tonic conductance of extrasynaptic GABA_A_ receptors is considered critical to producing the shunting inhibition of neuronal network excitability and, given this capability, it is reasoned to be a target mechanism for tinnitus pharmacology (21). Brexanolone, a molecule chemically identical to the neuroactive steroid allopregnanolone, is a potent positive allosteric modulator of both synaptic and extrasynaptic GABA_A_ receptors (24) and, thus, has the potential to enhance both phasic and tonic inhibition in the brain. Following this rationale, we conducted a proof-of-mechanism study of brexanolone, which is the first clinical trial to investigate the utility of a novel neuroactive steroid mechanism of tinnitus management.

## Materials and methods

### Design and sample

This phase 2, single-arm, open-label pilot study (NCT05645432) enrolled 18- to 65-year-old adults diagnosed with chronic, idiopathic, bilateral, non-pulsatile, and subjective tinnitus. This study was approved by a local institutional review board and performed in accordance with ethical principles that have their origin in the Declaration of Helsinki; all participants provided informed consent. Participant recruitment began on 10 May 2023, with both recruitment and study completion occurring on 21 November 2023. Originally, a record of a formal diagnosis was required to have been obtained from at least 6 months to no more than 5 years prior to the start of the study; the chronicity criteria were later amended to allow for the enrollment of participants with a diagnosis from up to 10 years prior to beginning of the study. These evaluations were conducted by audiologic professionals outside of the study protocol using standard clinical procedures. During screening, participants were required to have mild to moderate tinnitus symptom severity at screening, defined as a score of 24–68 on the Tinnitus Handicap Inventory (scale range 0 to 100) (25); the use of imaging was not required to exclude confounding conditions, such as retrocochlear or intracranial lesions. Participants were further required to safely discontinue the use of central nervous system (CNS) depressants (e.g., opioids and benzodiazepines), antidepressants, anticonvulsants, CNS stimulants (with the exception of caffeine), aspirin, other nonsteroidal anti-inflammatory drugs, and aminoglycosides at least 14 days or five half-lives (whichever was longer) prior to the baseline assessment, before receiving brexanolone, and through completion of the study. Prior treatment for tinnitus was not exclusionary; however, participants were excluded if they intended to start or discontinue a pharmacological or nonpharmacological therapy (e.g., psychotherapy, sound therapy, masking, transcranial magnetic stimulation) for tinnitus during the study or if they had exposure to another investigational drug or device within 30 days or five half-lives of the investigational drug (whichever was longer) prior to the visit on day 1. Participants could not have a history of neurological disease or other chronic health condition that could account for tinnitus. Participants were given a full audiological examination, which included a pure-tone audiogram, a physical examination with movement tests to evaluate whether tinnitus could be attributed to a somatosensory cause, and the intake of clinical history to evaluate duration and quality of tinnitus. Participants were excluded from the study if they had current unilateral or bilateral hearing loss of 30 dB or greater (mild hearing loss) in one or more tested frequencies (500 Hz, 1,000 Hz, 2,000 Hz, and 4,000 Hz), hearing loss of 60 dB or greater at 6,000 Hz and 8,000 Hz, asymmetry of 30 dB or greater in two or more tested frequencies, or if they used a cochlear implant or hearing aid. We aimed to enroll 20 participants.

### Dosing and administration

A single 6-h intravenous infusion of brexanolone was administered at an inpatient facility as a 90 mcg/kg/h infusion dose for 5 h following a 1-h titration period (0–30 min at 30 mcg/kg/h, 30–60 min at 60 mcg/kg/h) monitored by medical staff. This dose was selected to allow adequate time to approach steady-state levels of brexanolone and to rapidly achieve plasma concentrations of brexanolone in the range experienced during therapy for its approved use to treat postpartum depression. Moreover, 90 mcg/kg/h given as a short-term infusion (i.e., 4 h) has previously been well tolerated in an unpublished brexanolone pharmacokinetic/mass balance study. Brexanolone concentrations were expected to elicit target engagement following this dosing paradigm based on modeling by Wald et al. (26).

### Procedures

Screening occurred up to 28 days prior to dosing. Vital signs, Columbia–Suicide Severity Rating Scale (C-SSRS) (27), Patient Health Questionnaire (PHQ-9) (28), Clinical Global Impression-Severity (CGI-S) (29), Tinnitus Handicap Inventory (THI) (30), Tinnitus Functional Index (TFI) (31, 32), and audiology diagnostics were administered at screening.

From day-7 through day-1 prior to dosing, the visual analogue scale of tinnitus loudness (VAS-L) and annoyance (VAS-A) (33) were administered remotely two times daily in the morning and in the evening. The VAS-L scale asked participants to answer the question, “How loud is your tinnitus now?” rated on a horizontal scale anchored on the left by “not audible” (score of 0) and on the right by “extremely loud” (score of 100). For the VAS-A, participants were asked, “How much is your tinnitus annoying you now?” and responded using a horizontal scale anchored on the left by “not annoying” (score of 0) and on the right by “extremely annoying” (score of 100).

Prior to dosing on day 1, VAS-L, VAS-A, and tinnitus loudness matching (34) were rated. Perceived tinnitus loudness was assessed using an audiometric testing procedure; tinnitus loudness matching was conducted on MedRx’s Tinnometer device by following the manufacturer’s instructions (35). The procedure involved first modulating a tone by frequency and waveform to most closely match the perceived tinnitus. The volume of the matched tone was then adjusted by the participant to equal the perceived intensity of their tinnitus. Frequency matching was only conducted at baseline, and the same test stimuli were administered to measure intensity matching at the post-infusion and end-of-study (EOS) examinations. During the infusion, VAS-L and VAS-A were rated at 0.5, 1, 2, 3, 4, 5, and 6 h.

Sedation was monitored throughout the infusion using the Modified Observer’s Assessment of Alertness Scale (36). Safety was monitored via clinical labs (including renal and hepatic biochemistry panels, hematology, and urinalysis), vital signs, pulse oximetry, electrocardiograms, physical findings, and other observations related to safety. Adverse events (AEs) were monitored throughout treatment and follow-up. Blood samples for pharmacokinetic analysis were collected pre-dose and at 0.5, 1, 2, 3, 4, 5, 6, and 8 h post-dose timepoints. Plasma concentrations of brexanolone were determined using a fully validated liquid chromatography tandem mass spectrometry (LC–MS/MS) method with a deuterated brexanolone-d_4_ as the internal standard. The plasma samples were subjected to liquid–liquid extraction with methyl-tert-butyl-ether (MTBE) and reconstituted with methanol:water:formic acid (50:50:0.05). Processed samples were chromatographically separated on an ACE Excel C18 (50 × 2.1 mm, 2 μM) column (Mac-Mod Analytical, Inc.) with high-performance liquid chromatography-grade methanol and water (0.1% formic acid) as the mobile phase. Mass spectrometric analysis was performed on a SCIEX API 4500 mass spectrometer equipped with an electrospray ionization source in positive mode (ESI+). The ESI source settings were as follows: IonSpray voltage, 5,500 V; source temperature, 600 °C; collision gas, 8 AU; curtain gas, 30 AU; and nebulizer gas pressure, 50 bar. Multiple reaction monitoring (MRM) transitions and related collision energies were m/z 319.2 > 283.3 (21 V) for brexanolone and 323.3 > 287.3 (21 V) for brexanolone-d_4_. The linear range of the validated assay was 1–500 ng/mL.

Participants rated VAS-L and VAS-A in diaries at home two times a day for 6 days following dosing. Participants returned to the clinic on day 7 ± 1 for an EOS visit and were administered follow-up assessments using the C-SSRS, PHQ-9, CGI-S, CGI change (CGI-C), VAS-L, VAS-A, THI, and TFI scales, as well as audiometric testing of tinnitus loudness matching at the frequency identified during baseline assessment.

The THI was first published as a three-labeled category scale; however, for this study, numeric values were applied, and the test was scored following the standard set by McCombe et al. (25). The subscales of “Catastrophic,” “Emotional,” and “Functional” were reported for the THI along with the summed “Total Score.” The subscales of “Auditory,” “Cognitive,” “Control,” “Emotional,” “Intrusive,” “Quality of Life,” “Relaxation,” and “Sleep” were reported for the TFI along with a summed “Total Score.” Published values for the minimally clinically important difference (MCID) were used as a reference for the interpretation of change observed on standard tinnitus instruments as follows: 10–15 points in tinnitus loudness or annoyance on the 100-point VAS-L and VAS-A (33), 7 points for the THI total score (37), 7 points for the TFI total score (38), and 5 dB in tinnitus loudness matching (39).

### Experimental design and statistical analyses

Descriptive statistics were calculated and summarized for all consented participants who received brexanolone. Primary outcomes were safety and tolerability measures, such as treatment-emergent AEs (TEAEs), defined as any AE (new or worsening from baseline) with an onset at or after the initiation of the infusion. Secondary outcomes were changes from baseline on VAS-L and VAS-A on day 1, as well as between days 2 and 7. Data collected using all additional clinical and audiometric procedures were considered exploratory.

Assessments completed on the dosing day (day 1) were meant to assess acute effects, whereas assessments completed on following days (days 2–7) were meant to assess the durability of the effect; therefore, the selection of “baseline” measurement differed for day 1 and day 2–7 assessments. For the VAS assessments completed at the dosing visit (day 1), baseline was defined as the most recent pre-dose value immediately prior to the start of brexanolone infusion. For the twice-daily VAS assessments completed following dosing (days 2 through 7), baseline was defined as the average of all available pre-infusion values for the participant collected from days 7 to 1. This was calculated by summing the daily arithmetic means and dividing by the total number of non-missing days. If a participant missed either a morning or evening score, then the single available score was used as the mean for that day.

Analyses of change from baseline in VAS-L and VAS-A scores were performed separately using a linear mixed-effects model for repeated measures to assess the effect size of brexanolone in reducing tinnitus loudness and annoyance. Each model included the baseline value and post-baseline timepoints as fixed effects. Within-participant residual errors across timepoints were modeled using an unstructured covariance matrix. The results are reported as least squares (LS) means with corresponding 95% confidence intervals (CI), standard errors (SE), and *p*-values. For day 1, repeated timepoints were by hours, and for days 2–7, by days.

**Figure 1:**
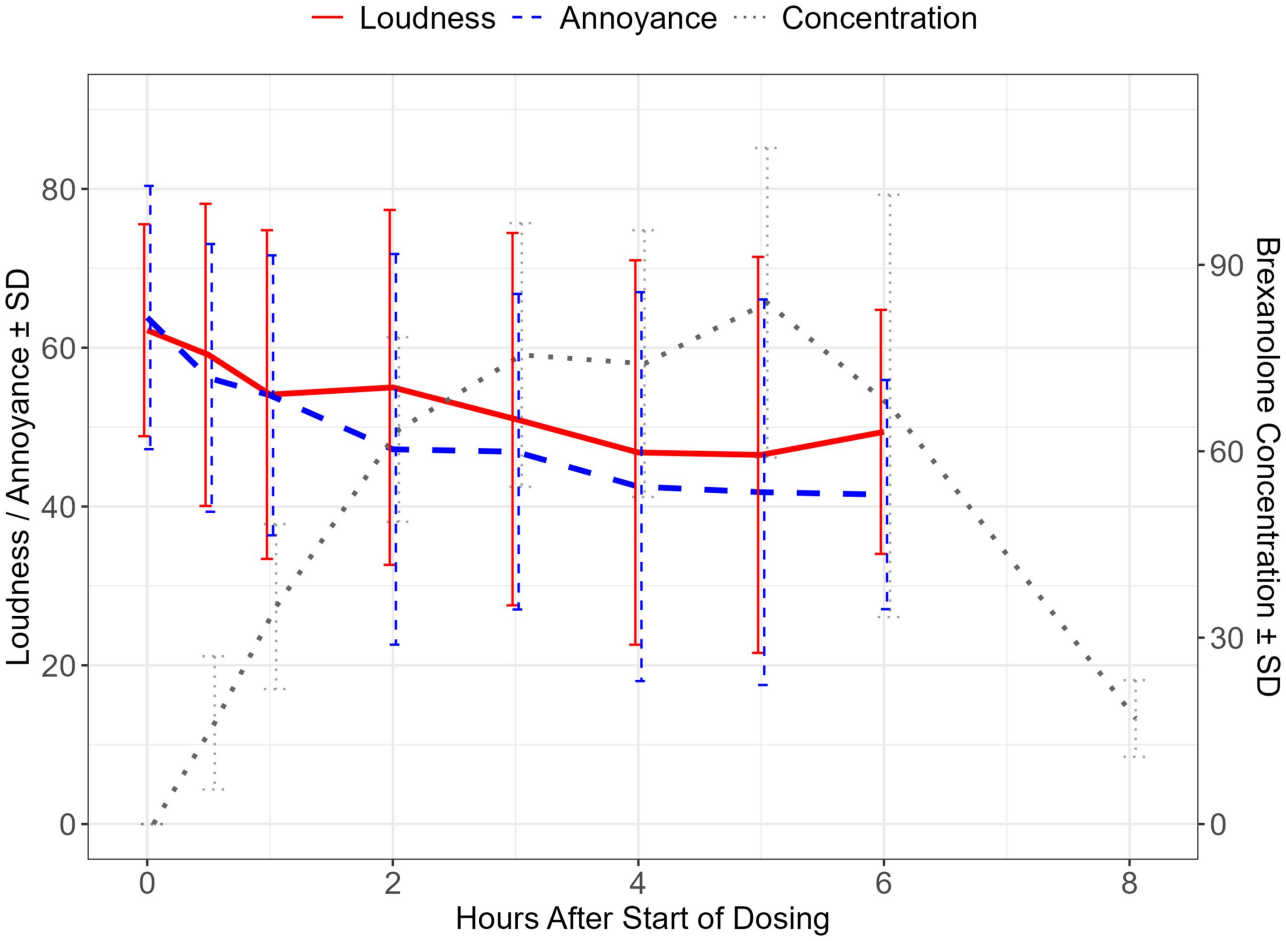
Brexanolone concentration and change scores for VAS-L and VAS-A during the infusion period. Mean ± SD of visual analogue scale for loudness (VAS-L) and annoyance (VAS-A) scores during the brexanolone infusion period, depicted on the left y-axis. Mean ± standard deviation of brexanolone concentrations over the infusion period plus one post-dose timepoint, depicted on the right y-axis. SD, standard deviation; PK, pharmacokinetic data point.

**Figure 2:**
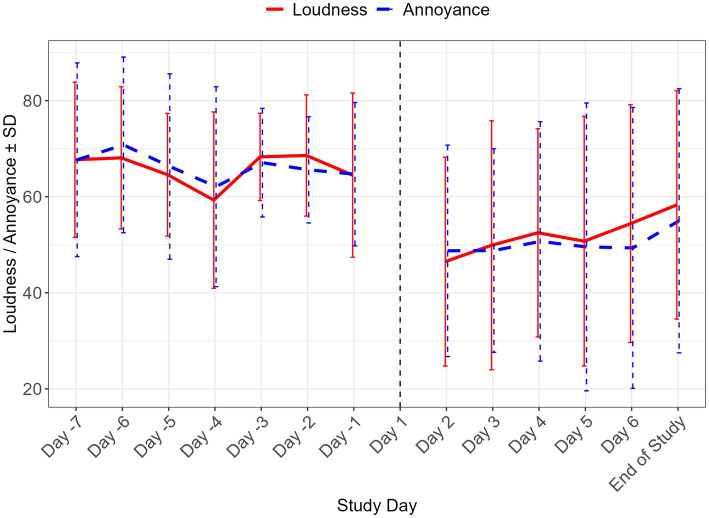
Change in VAS-L and VAS-A scores over the post-infusion week. Mean ± SD of visual analogue scale loudness (VAS-L) and annoyance (VAS-A) scores during the pre-infusion week and in the post-infusion period. Day 1 indicates dosing day. SD, standard deviation.

The effect size at each post-baseline time point was calculated relative to the baseline score using the unbiased Hedge’s *g* method, which adjusts for small sample size. Effect sizes of 0.2, 0.5, and 0.8 were considered small, medium, and large, respectively (40). Exploratory analyses were carried out to examine changes in audiometric measurement of tinnitus loudness matching from pre-dose to post-dose (day 1) and to EOS, as well as changes between screening and EOS in THI, TFI, and CGI-S. All analyses were performed using SAS^®^ software version 9.4 (41). Figures 1, 2 were generated using the ggplot2 package in R version 4.2.2 (42, 43).

## Results

### Study participants

A total of 10 of the 29 screened participants met the inclusion criteria and enrolled in the study between May and November of 2023. All 10 participants completed dosing; however, one participant withdrew from the study during the follow-up period for reasons unrelated to brexanolone administration. Out of the target sample (*n* = 20), enrollment ended after the first 10 participants completed all procedures (Table 1). This was due to factors influencing the study timeline, among other corporate priorities. Given the consistency of data supporting a positive trend in the treatment response within this probe trial, the completed portion of the sample was considered sufficient to answer the questions that inspired the study. Ten participants completed all baseline assessments, as well as both the infusion-period VAS scoring and post-infusion tinnitus loudness matching. Nine participants completed the EOS measures.

**Table 1:** Baseline characteristics and tinnitus medical history of efficacy population.

Characteristic	*n* = 10
Participant demographics	
Age in years, mean (SD)	48.8 (9.96)
Sex, *n* (%)	
Male	3 (30%)
Female	7 (70%)
Race, *n* (%)	
Black or African American	4 (40%)
White	6 (60%)
Ethnicity, *n* (%)	
Not Hispanic or Latino	10 (100%)
Height in cm, mean (SD)	170.9 (14.86)
Weight in kg, mean (SD)	94.3 (19.77)
BMI in kg/m^2^, mean (SD)	32.4 (5.98)
Subjective tinnitus history	
Years with tinnitus diagnosis^a^, mean (range)	4.6 (1–9)
Initial tinnitus onset relationship, *n* (%)	
Loud blast of sound	1 (10%)
Unknown etiology	9 (90%)
Consistency of tinnitus loudness, *n* (%)	
Fairly constant day-to-day	8 (80%)
Fluctuates widely	2 (20%)
Percent total awake time aware of tinnitus, mean (SD)	77.3 (15.19)
Percent total awake time annoyed, distressed, or irritated by tinnitus, mean (SD)	72.3 (19.60)

cm, centimeters; kg, kilogram; m^2^, square meters; n, sample size; SD, standard deviation.

^a^Length of time from a formal diagnosis.

### Change from baseline in visual analogue scales—loudness and annoyance

#### Visual analogue scale–loudness (VAS-L)

The impact of brexanolone on tinnitus loudness was assessed using the VAS-L scale. At day 1 (pre-dose) baseline, mean ± SD loudness on the VAS-L was reported as 62.2 ± 13.35. The model-based LS mean change from baseline reached a proposed threshold for the MCID (33) by hour 3 (LS mean ± SE: −11.2 ± 4.47, 95% CI: −21.35, −1.05; *p* = 0.03), and this magnitude of difference was maintained at all subsequently measured timepoints including at the end of the 6-h infusion (−12.8 ± 3.01, 95% CI: −19.64, −5.96; *p* < 0.01). At hour 6 (end of infusion), the effect size for the change from baseline calculated using Hedge’s *g* was −0.84 (95% CI: −1.33, −0.35) (Tables 2, 3 and Figure 1).

**Table 2:** VAS scale scores during brexanolone dosing day and in the week following dosing.

	VAS-loudness		VAS-annoyance	
	Mean (SD)	Hedges *g*^a^	Mean (SD)	Hedges *g*^a^
Dosing day				
Day 1 - Baseline	62.2 (13.35)		63.8 (16.58)	
0.5 h	59.1 (19.03)	−0.14	56.2 (16.86)	−0.44
1 h	54.1 (20.70)	−0.40	54.0 (17.63)	−0.55
2 h	55.0 (22.35)	−0.31	47.2 (24.60)	−0.76
3 h	51.0 (23.45)	−0.44	46.9 (19.87)	−0.88
4 h	46.8 (24.21)	−0.61	42.5 (24.49)	−0.97
5 h	46.5 (24.94)	−0.56	41.8 (24.28)	−1.01
6 h	49.4 (15.37)	−0.84	41.5 (14.43)	−1.37
Comparison of pre- and post-dosing weeks				
Average pre-dose week	66.3 (11.57)		65.9 (13.77)	
Day 2	48.8 (21.72)	−0.90	46.5 (22.00)	−0.97
Day 3	48.8 (25.92)	−0.74	49.9 (21.19)	−0.84
Day 4	50.7 (21.68)	−0.82	52.5 (24.92)	−0.61
Day 5	49.6 (26.00)	−0.71	50.8 (29.96)	−0.56
Day 6	49.4 (24.74)	−0.77	54.4 (29.24)	−0.45
End of Study	55.0 (23.77)	−0.51	58.3 (27.49)	−0.31

SD, standard deviation; VAS, visual analogue scale.

^a^Results > |0.5| are considered moderate, and > |0.8| are considered large based on Cohen’s interpretation of Hedge’s *g* (40).

**Table 3:** Least squares mean change from baseline in VAS scale scores.

	Statistic	VAS-loudness	VAS-annoyance
Dosing day			
Day 1 – Overall	LS Means (SE)	−10.5 (3.64)	−16.6 (5.90)
	95% CI	(−18.76, −2.24)	(−30.09, −3.19)
	*P*-value	0.0184	0.021
0.5 h	LS Means (SE)	−3.1 (2.49)	−7.6 (4.09)
	95% CI	(−8.81, 2.61)	(−17.01, 1.81)
	*P*-value	0.2468	0.0996
1 h	LS Means (SE)	−8.1 (4.44)	−9.8 (5.19)
	95% CI	(−18.15, 1.95)	(−21.58, 1.98)
	*P*-value	0.1015	0.0924
2 h	LS Means (SE)	−7.2 (4.47)	−16.6 (8.11)
	95% CI	(−17.33, 2.93)	(−35.07, 1.87)
	*P*-value	0.1421	0.0723
3 h	LS Means (SE)	−11.2 (4.47)	−16.9 (5.79)
	95% CI	(−21.35, −1.05)	(−30.01, −3.79)
	*P*-value	0.0342	0.0171
4 h	LS Means (SE)	−15.4 (5.04)	−21.3 (7.98)
	95% CI	(−26.82, −3.98)	(−39.45, −3.15)
	*P*-value	0.0139	0.0265
5 h	LS Means (SE)	−15.7 (4.83)	−22.0 (7.88)
	95% CI	(−26.66, −4.74)	(−39.93, −4.07)
	*P*-value	0.0103	0.0217
6 h	LS Means (SE)	−12.8 (3.01)	−22.3 (5.17)
	95% CI	(−19.64, −5.96)	(−34.56, −10.04)
	*P*-value	0.0022	0.0036
Comparison of pre- and post-dosing weeks			
Pre- to post-week – Overall	LS Means (SE)	−16.0 (7.25)	−13.9 (7.93)
	95% CI	(−33.30, 1.40)	(−33.54, 5.84)
	*P*-value	0.066	0.1343
Day 2	LS Means (SE)	−17.6 (6.77)	−19.4 (7.27)
	95% CI	(−33.76, −1.36)	(−38.44, −0.38)
	*P*-value	0.0376	0.0471
Day 3	LS Means (SE)	−17.5 (8.04)	−16.0 (8.11)
	95% CI	(−36.64, 1.63)	(−37.65, 5.64)
	*P*-value	0.0671	0.1125
Day 4	LS Means (SE)	−15.6 (6.81)	−13.4 (8.29)
	95% CI	(−31.57, 0.35)	(−33.97, 7.16)
	*P*-value	0.0541	0.1597
Day 5	LS Means (SE)	−16.8 (8.06)	−15.2 (8.72)
	95% CI	(−35.93, 2.41)	(−35.45, 5.14)
	*P*-value	0.0773	0.1223
Day 6	LS Means (SE)	−17.0 (7.72)	−11.5 (9.52)
	95% CI	(−35.08, 1.16)	(−34.14, 11.13)
	*P*-value	0.0627	0.2671
End of study	LS Means (SE)	−11.3 (7.33)	−7.6 (8.08)
	95% CI	(−29.24, 6.63)	(−26.78, 11.56)
	*p*-value	0.1739	0.3783

CI, confidence interval; LS, least squares mean; SE, standard error; VAS, visual analogue scale.

Next, we assessed the durability of VAS-L change during the week following infusion. The mean (SD) VAS-L score for the pre-dose week was 66.3 ± 11.57. Model-based LS mean ± SE changes from baseline ranged from −17.6 ± 6.77 (95% CI: −33.76, −1.36; *p* = 0.04) on day 2 through −11.3 ± 7.33 (−35.08, 1.16; *p* > 0.10) at EOS and were considered to be clinically meaningful according to the proposed MCID criteria. At EOS, the effect size (Hedge’s *g*) for the change from baseline was −0.51 (95% CI: −1.15, 0.13) (Tables 2, 3 and Figure 2).

#### Visual analogue scale–annoyance (VAS-A)

Concurrently with VAS-L assessments, participants rated tinnitus annoyance using the VAS-A scale. At baseline, the mean ± *SD* VAS-A was 63.8 ± 16.58. Model-based LS mean change from baseline exceeded the proposed MCID at 2 h after the start of infusion (LS mean ± SE: −16.6 ± 8.11, 95% CI: −35.07, 1.87; *p* = 0.07) and was maintained at all subsequently measured timepoints, including at the end of the infusion (−22.3 ± 5.17, 95% CI: −34.56, −10.04; *p* < 0.01). At hour 6, the effect size (Hedge’s *g*) for the change from baseline was −1.37 (95% CI: −2.63, −0.12) (Tables 2, 3 and Figure 1).

The durability of change in VAS-A was also assessed in the week following infusion. The mean ± *SD* VAS-A score for the pre-dose week was 65.9 ± 13.77. For the post-dosing day comparisons, model-based LS mean ±SE changes from baseline ranged from −19.4 ± 7.27 (95% CI: −38.44, −0.38, *p* = 0.05) on day 2 to −7.6 ± 8.08 (95% *CI*: −26.78, 11.56; *p* > 0.10) at EOS. At EOS, the effect size (Hedge’s *g*) for the change from baseline was −0.31 (95% *CI:* −0.97, 0.36) (Tables 2, 3 and Figure 2).

### Change from baseline in tinnitus handicap inventory

The THI assessed the impact of brexanolone on tinnitus-related mental, social/occupational, and physical functioning (25). At baseline (*n* = 10), the mean ± SD THI overall score was 50.8 ± 12.41 (range: 28–68). At EOS (*n* = 9), the score decreased to 39.8 ± 15.89 (range: 14–70), with a mean change from baseline of −12.0 ± 15.84, exceeding the proposed MCID threshold (37). Hedge’s *g* at EOS was −0.79 (95% *CI*: −1.67, 0.09) (Table 4).

**Table 4:** Results for tinnitus handicap inventory and tinnitus functional index pre- and post-brexanolone infusion.

Test	Baseline	End of study	
	Mean (SD)	Mean (SD)	Hedge’s *g*^a^
THI total	51.8 (12.7)	39.8 (15.9)	0.75
Catastrophic	11.3 (4.12)	10.7 (4.69)	0.14
Emotional	16.4 (5.46)	12.4 (7.54)	0.53
Functional	24.0 (8.06)	16.7 (6.08)	0.89
TFI total	69.9 (12.6)	56.2 (15.9)	0.86
Auditory	65.6 (22.4)	40.4 (29.8)	0.86
Cognitive	66.7 (15.4)	58.1 (24.2)	0.37
Control	77.4 (22.2)	74.8 (13.8)	0.11
Emotional	61.5 (16.8)	44.8 (30.1)	0.58
Intrusive	81.1 (10.7)	71.9 (11.9)	0.74
QoL	62.2 (23.6)	39.7 (20.7)	0.91
Relaxation	77.8 (14.1)	68.5 (23.3)	0.39
Sleep	69.6 (23.9)	56.7 (25.5)	0.47

TFI, tinnitus functional index; THI, tinnitus handicap inventory; QoL, quality of life; SD, standard deviation.

^a^Results > |0.5| are considered moderate, and > |0.8| are considered large based on Cohen’s interpretation of Hedge’s *g* (40).

### Change from baseline in tinnitus functional index

The TFI is sensitive to changes in the functional effects of tinnitus (44); hence, we aimed to explore the impact of brexanolone treatment on TFI scores. At baseline (*n* = 10), the mean ± SD TFI total score was 67.5 ± 14.01 (range: 46.4–84.4). At EOS (*n* = 9), the score decreased to 56.2 ± 15.95 (range: 32.0–77.6), with a mean change from baseline of −13.7 ± 18.68, exceeding based on the proposed MCID threshold (38). Hedge’s *g* at EOS was −0.91 (95% *CI*: −1.99, 0.18) (Table 4).

### Change from baseline in tinnitus loudness matching (pure-tone audiometry)

To assess the change in perceived loudness of tinnitus following the infusion period, audiometric testing was employed. At baseline (prior to infusion), the mean ± SD relative tinnitus loudness was 10.2 ± 5.88 dB in the right ear and 14.0 ± 11.29 dB in the left ear. After the infusion period, there was a − 3.4 ± 7.21 dB and a − 6.4 ± 12.96 dB change from baseline in the right and left ears, respectively. The effect sizes (Hedge’s *g*) for these changes were −0.55 (95% *CI*: −1.42, 0.31) and −0.65 (−1.66, 0.35) for the right and left ears, respectively (*n* = 10 for these analyses).

To test the durability of effects, tinnitus loudness was measured at the EOS visit, where the changes from baseline (*n* = 9) were −1.9 ± 7.77 dB and a − 7.6 ± 12.70 dB in the right and left ears, respectively, representing effect sizes (Hedge’s *g*) of −0.36 (95% *CI*: −1.48, 0.76) and −0.83 (95% *CI*: −2.04, 0.37), respectively (Table 5). The reduction in perceived intensity exceeded published MCID thresholds (39), indicating meaningful change for the left ear only.

**Table 5:** Comparison of mean relative tinnitus loudness pre-dose, post-dose, and at end of study.

Relative tinnitus loudness					
	Pre-dose	Post-dose		End of study	
	Mean (SD)	Mean (SD)	Hedge’s *g*^a^	Mean (SD)	Hedge’s *g*^a^
Right ear	10.2 (5.88)	6.8 (5.92)	−0.55	8.9 (3.95)	−0.36
Left ear	14.0 (11.29)	7.6 (6.88)	−0.65	7.4 (4.53)	−0.83

SD, standard deviation. Pre-dose and post-dose sample size *n* = 10; EOS visit sample size was *n* = 9.

^a^Results > |0.5| are considered moderate, and > |0.8| are considered large based on Cohen’s interpretation of Hedge’s *g* (40).

Interpretation of effects on tinnitus loudness matching according to the proposed MCID guidance (39) was considered mixed, given that the reduction in perceived loudness (decibels, dB) exceeded the recommended for left ear measurements only.

### Clinical global impression scales

At the EOS visit, CGI-C scale ratings were reported as minimally improved for 3 (30%) participants, and much improved, no change, or minimally worse for 2 (20%) participants each.

### Safety

A single, mild treatment-emergent adverse event (TEAE) of tinnitus (reported term: worsening tinnitus) was reported at the safety follow-up assessment on day 15 following brexanolone administration, 8 days after the participant’s EOS visit. The event was recorded for completeness but was not considered by the investigator to be related to brexanolone treatment. There were no clinically meaningful mean changes from baseline through EOS in clinical laboratory parameters, vital signs, or electrocardiogram findings. One participant had two decreases in systolic blood pressure (SBP) that met protocol criteria for clinical significance (≥30 mmHg SBP change from baseline) but were not accompanied by clinical symptoms and were not reported as AEs. This participant’s systolic blood pressure (SBP) was 136 mm Hg at baseline, decreased to 103 mm Hg at hour 3, 106 mm Hg at hour 4, and returned to 139 mm Hg (similar to baseline) at hour 5.

### Pharmacokinetics

The geometric mean T_max_ for brexanolone was 6.04 (%coefficient of variation (CV) = 0.398) h. The geometric mean C_max_ was 78.1 (%CV = 25.6%) ng/mL and the geometric mean area under the concentration vs. time curve from time 0 to 6 h was 362 (%CV = 29.1) ng * hour / mL. Brexanolone concentrations for each time point are depicted in Figure 1 and Supplementary Table 1.

## Discussion

The current study was the first to evaluate the potential of brexanolone, a drug analog of the endogenous neuroactive steroid allopregnanolone that has balanced affinity at synaptic and extrasynaptic GABA_A_ receptors, for the management of tinnitus symptomatology. In this proof-of-mechanism study, brexanolone was observed to be safe and well-tolerated at the planned dose and duration by adult participants with tinnitus. Additionally, preliminary support for the hypothesized effect of brexanolone in the reduction of tinnitus experience in a small group of 10 participants with moderate tinnitus was obtained. Rapid and sustained reductions in measures of perceived tinnitus severity (Figures 1, 2 and Tables 2–4) were observed in the context of a lessening burden of tinnitus on activities of daily living (Table 4). Specifically, an exposure-dependent reduction in subjective tinnitus severity was observed over a single 6-h infusion of brexanolone, with progressive reduction in VAS ratings of loudness and annoyance observed over 1-h increments of monitoring. Change in VAS scores coincided with a reduction in tinnitus loudness matching, as measured by an audiometric testing procedure, lending confidence that changes in subjective ratings reflected detectable differences in psychoacoustic properties of tinnitus experience. Importantly, reductions in both VAS ratings and volume matching persisted for 1 week post-infusion and were accompanied by improvement on standard tinnitus severity indexes, including the THI and TFI. The exposures achieved in tinnitus patients (Supplementary Table 1) were sufficient to test the hypothesis that balanced GABA positive allosteric modulator activity can play a role in the treatment of tinnitus.

There is compelling evidence linking tinnitus to a disruption of excitatory and inhibitory inputs within the central auditory pathways. The GABA_A_ receptor is highly expressed in the auditory system (14), and GABAergic drugs, including the benzodiazepine derivatives, alprazolam (8) and clonazepam (9), and the GABA homolog, gabapentin (10, 12), were the focus of early exploration of pharmacological intervention. However, these medications have not demonstrated a robust evidence base for efficacy in tinnitus trials (21, 45), and the American Academy of Otolaryngology has further discouraged clinical use due to the unfavorable risk–benefit profile of benzodiazepines (46), acknowledging both inconsistent effects in randomized, placebo-controlled trials and risks such as abuse liability, dependence, and sedation (2, 45). The emergence of novel GABAergic drugs, distinguished both by mechanistic action and a balanced binding affinity at both synaptic and extrasynaptic GABA_A_ receptors, offers a new opportunity to revisit the GABA hypothesis of tinnitus and extend converging evidence from neurochemical, anatomical, physiological, and pharmacological perspectives to clinical investigation (21). The results of the current study provide the first clinical evidence to support the hypothesis that drugs with the potential to engage extrasynaptic, in addition to synaptic, GABA_A_ receptors offer a promising new pathway for drug development in tinnitus. This hypothesis is based, in part, on the potential of these drugs to enhance tonic inhibition, which regulates network-level activity in the auditory system—a property that distinguishes neuroactive steroids from GABAergic drugs that bind selectively at synaptic sites.

Considered in the context of the extant literature on clinical trial outcomes in tinnitus, we find these results encouraging. Positive treatment responses are uncommon in tinnitus clinical trials. A meta-analysis of treatment effect sizes compiled by Witkin et al. (21) presents a near equivalent balance in the number of tested drug mechanisms that have resulted in improving and worsening symptoms relative to placebo. Where improvement has been observed, effects are generally modest, with a standardized mean difference (SMD) of 1.0 achieved by only a single mechanism (amitriptyline), followed by 3 effects in the *SMD* = 0.5–0.8 range (acamprosate, gabapentin + lidocaine, dexamethasone + melatonin). Notably, the standardized effect for clonazepam in this meta-analytic review was −0.01 (95% *CI =* −1.17, 1.16), with gabapentin alone fairing only slightly better (*SMD* = −0.16, 95% *CI* = −0.64, 0.31); thus, neither mechanism offered confident support for GABAergic modulators as a stand-alone treatment. The current study evaluated change from baseline in outcome measures using Hedge’s *g*, lending further interpretability of change indices in terms of standardized effect sizes. On VAS measures of tinnitus loudness and annoyance, large effects (> 0.80) were obtained at the end of infusion relative to pre-infusion baseline. When evaluated as a measure of average change aggregated over week-long pre-dose baseline and follow-up periods, medium effects were observed (> 0.50), with attenuation of the initial response observed on later days of the follow-up period (Figure 2); however, VAS ratings remained well below baseline (improved) at all measured timepoints during the follow-up period and were accompanied by medium-large effects on TFI and THI at EOS (Table 4), suggesting improvement in activities of daily living. Audiometric measures of tinnitus volume matching provided a complementary assessment of reduction in tinnitus intensity from pre- to post-infusion periods on day 1, with medium effect sizes for right and left ear measures. The volume matching procedure is commonly administered unilaterally using the side on which tinnitus is loudest, and bilateral testing in our study yielded a discrepant pattern of response from post-infusion to the end of follow-up, with a larger effect size on the left side (large effect size, Table 5).

Although no placebo control was used in the current study, it should be noted that several trials reported no appreciable change from baseline in their placebo arms on the same outcome measures used in this study: Johnson et al. (8) for VAS and loudness matching; Jalali et al. (47) for VAS, loudness matching, and THI; and Bahmad et al. (9) for VAS. Moreover, although effect sizes varied according to outcome measure and by time of assessment relative to brexanolone dosing (post-infusion, EOS), it is important to also note that measured outcomes consistently showed improvement and exceeded published MCID thresholds, indicating clinically meaningful change for the VAS (33), THI total score (37), TFI total score (38), and for tinnitus loudness matching audiometry (39).

As this was a small, single-arm, single-infusion, open-label, proof-of-mechanism study, results should be interpreted with caution. Although our trial was designed to investigate the potential of brexanolone to attenuate tinnitus symptomatology, alternative explanations for change observed across outcome measures over the trial period cannot be ruled out, including placebo effect, the influence of participants’ expectation bias, or other non-specific effects of clinical attention and study-related activity. Apart from the absence of a placebo control, it is important to recognize several other limitations in our study design that may affect the generalizability of our results. First, the sample size was small and excluded participants with very mild or very severe tinnitus at baseline; nonetheless, the moderate to large Hedge’s *g* effect sizes observed across various outcome measures are encouraging. Second, the 7-day follow-up period limited the evaluation of long-term durability of the initial treatment response. Attenuation of initial effects became evident toward the end of the follow-up period, particularly on the VAS measures, and suggests that repeat treatment might be necessary for clinical management. Indeed, both the durability of effect and potential for complications should be investigated following longer dosing and a more substantial follow-up period. Finally, although the use of intravenous administration was considered appropriate for this proof-of-mechanism study, this administration method may not be feasible for drugs intended for routine clinical management of tinnitus. Of note, the sponsor (Sage Therapeutics) does not intend to develop brexanolone for tinnitus treatment but used this particular compound to test a hypothesis that could lead to the development of orally bioavailable compounds optimized for synaptic and extrasynaptic GABA_A_ activity.

## Conclusion

Brexanolone was well tolerated and showed no prohibitive safety findings within the parameters of use in this single-infusion trial of adults with tinnitus. Overall, clinically meaningful improvements relative to baseline were observed in subjective perception of tinnitus loudness and annoyance within hours of initiating brexanolone infusion; these improvements were extended to a reduction in audiometric measures of tinnitus loudness and participant-reported functional improvements measured 1 week following the infusion. Contrasted with results of clinical trials evaluating the efficacy of benzodiazepine drugs, the rapidity and duration of response to brexanolone in our open-label trial are encouraging, especially when weighed against the lack of robust placebo response observed in double-blind drug trials. Accordingly, the present study provides initial clinical support for the hypothesis that extrasynaptic GABA_A_ modulation could provide treatment for some forms of tinnitus.

## Data Availability

All relevant data is contained within the article. The original contributions presented in the study are included in the article/supplementary material, further inquiries can be directed to the corresponding author.

## Supplementary material

SUPPLEMENTARY FIGURE 1 Diagram of study selection.

SUPPLEMENTARY FIGURE 2 Data from the analysis of VAS loudness **(A)** and annoyance **(B)** over the infusion period. Mean is represented by the middle open circle, median as the line, and the interquartile ranges as whiskers.

SUPPLEMENTARY FIGURE 3 Data from the analysis of VAS loudness **(A)** and annoyance **(B)** over the post-dosing week. Mean is represented by the middle open circle, median as the line, and the interquartile ranges as whiskers.

## References

[1] Bhatt JM Lin HW Bhattacharyya N. Prevalence, severity, exposures, and treatment patterns of tinnitus in the United States. JAMA Otolaryngol Head Neck Surg. (2016) 142:959–65. doi: 10.1001/jamaoto.2016.1700, PMID: 27441392 PMC5812683

[2] Dalrymple SN Lewis SH Philman S. Tinnitus: diagnosis and management. Am Fam Physician. (2021) 103:663–71. PMID: 34060792

[3] Han BI Lee HW Kim TY Lim JS Shin KS. Tinnitus: characteristics, causes, mechanisms, and treatments. J Clin Neurol Seoul Korea. (2009) 5:11–9. doi: 10.3988/jcn.2009.5.1.11, PMID: 19513328 PMC2686891

[4] Maihoub S Mavrogeni P Répássy GD Molnár A. Exploring how blood cell levels influence subjective tinnitus: a cross-sectional case-control study. Audiol Res. (2025) 15:72. doi: 10.3390/audiolres15030072, PMID: 40558411 PMC12190120

[5] Molnár A Molnár V Mavrogeni P Maihoub S. Fasting glucose, haemoglobin A1C (HbA1c), blood lipid, and triglyceride–glucose index parameters in relation to subjective tinnitus. Biomedicine. (2025) 13:824. doi: 10.3390/biomedicines13040824, PMID: 40299446 PMC12024736

[6] Cima RFF Mazurek B Haider H Kikidis D Lapira A Noreña A . A multidisciplinary European guideline for tinnitus: diagnostics, assessment, and treatment. HNO. (2019) 67:10–42. doi: 10.1007/s00106-019-0633-7, PMID: 30847513

[7] Tyler R Perreauf A Mohr A-M Ji H Mancini PC. An exploratory step toward measuring the “meaning of life” in patients with tinnitus and in Cochlear implant users. J Am Acad Audiol. (2020) 31:277–85. doi: 10.3766/jaaa.19022, PMID: 31580805

[8] Johnson RM Brummett R Schleuning A. Use of alprazolam for relief of tinnitus: a double-blind study. Arch Otolaryngol Head Neck Surg. (1993) 119:842–5. doi: 10.1001/archotol.1993.01880200042006, PMID: 8343245

[9] Bahmad FM Venosa AR Oliveira CA. Benzodiazepines and GABAergics in treating severe disabling tinnitus of predominantly cochlear origin. Int Tinnitus J. (2006) 12:140–4. 17260879

[10] Bauer CA Brozoski TJ. Effect of gabapentin on the sensation and impact of tinnitus. Laryngoscope. (2006) 116:675–81. doi: 10.1097/01.MLG.0000216812.65206.CD, PMID: 16652071

[11] Shulman A Strashun AM Goldstein BA. GABAA-benzodiazepine-chloride receptor-targeted therapy for tinnitus control: preliminary report. Int Tinnitus J. (2002) 8:30–6. 14763233

[12] Zapp JJ. Gabapentin for the treatment of tinnitus: a case report. Ear Nose Throat J. (2001) 80:114–6. doi: 10.1177/014556130108000211, PMID: 11233342

[13] Watanabe M Maemura K Kanbara K Tamayama T Hayasaki H. GABA and GABA receptors in the central nervous system and other organs In: International review of cytology: Elsevier (2002). 1–47. 10.1016/s0074-7696(02)13011-7 11837891

[14] Maison SF Rosahl TW Homanics GE Liberman MC. Functional role of GABAergic innervation of the cochlea: phenotypic analysis of mice lacking GABA_A_ receptor subunits α1, α2, α5, α6, β2, β3, or δ. J Neurosci. (2006) 26:10315–26. doi: 10.1523/JNEUROSCI.2395-06.2006, PMID: 17021187 PMC1806703

[15] Gao F Wang G Ma W Ren F Li M Dong Y . Decreased auditory GABA+ concentrations in presbycusis demonstrated by edited magnetic resonance spectroscopy. NeuroImage. (2015) 106:311–6. doi: 10.1016/j.neuroimage.2014.11.023, PMID: 25463460 PMC4285773

[16] Wang X Lu T Bendor D Bartlett E. Neural coding of temporal information in auditory thalamus and cortex. Neuroscience. (2008) 157:484–93. doi: 10.1016/j.neuroscience.2008.07.050, PMID: 19143093

[17] Yang S Weiner BD Zhang LS Cho S-J Bao S. Homeostatic plasticity drives tinnitus perception in an animal model. Proc Natl Acad Sci. (2011) 108:14974–9. doi: 10.1073/pnas.1107998108, PMID: 21896771 PMC3169130

[18] Sedley W Parikh J Edden RAE Tait V Blamire A Griffiths TD. Human auditory cortex neurochemistry reflects the presence and severity of tinnitus. J Neurosci. (2015) 35:14822–8. doi: 10.1523/JNEUROSCI.2695-15.2015, PMID: 26538652 PMC4635131

[19] Isler B Von Burg N Kleinjung T Meyer M Stämpfli P Zölch N . Lower glutamate and GABA levels in auditory cortex of tinnitus patients: a 2D-JPRESS MR spectroscopy study. Sci Rep. (2022) 12:4068. doi: 10.1038/s41598-022-07835-8, PMID: 35260698 PMC8904839

[20] Kim SH Kim D Lee J-M Lee SK Kang HJ Yeo SG. Review of pharmacotherapy for tinnitus. Healthcare. (2021) 9:779. doi: 10.3390/healthcare9060779, PMID: 34205776 PMC8235102

[21] Witkin JM Lippa A Smith JL Cook JM Cerne R. Can GABAkines quiet the noise? The GABAA receptor neurobiology and pharmacology of tinnitus. Biochem Pharmacol. (2022) 201:115067. doi: 10.1016/j.bcp.2022.115067, PMID: 35504315

[22] Richardson BD Brozoski TJ Ling LL Caspary DM. Targeting inhibitory neurotransmission in tinnitus. Brain Res. (2012) 1485:77–87. doi: 10.1016/j.brainres.2012.02.014, PMID: 22405692 PMC3374875

[23] Herd MB Foister N Chandra D Peden DR Homanics GE Brown VJ . Inhibition of thalamic excitability by 4,5,6,7-tetrahydroisoxazolo[4,5-c]pyridine-3-ol: a selective role for δ-GABA_A_ receptors. Eur J Neurosci. (2009) 29:1177–87. doi: 10.1111/j.1460-9568.2009.06680.x, PMID: 19302153 PMC2788206

[24] Althaus AL Ackley MA Belfort GM Gee SM Dai J Nguyen DP . Preclinical characterization of zuranolone (SAGE-217), a selective neuroactive steroid GABAA receptor positive allosteric modulator. Neuropharmacology. (2020) 181:108333. doi: 10.1016/j.neuropharm.2020.108333, PMID: 32976892 PMC8265595

[25] McCombe A Baguley D Coles R McKenna L McKinney C Windle-Taylor P. Guidelines for the grading of tinnitus severity: the results of a working group commissioned by the British Association of Otolaryngologists, head and neck surgeons, 1999. Clin Otolaryngol Allied Sci. (2001) 26:388–93. doi: 10.1046/j.1365-2273.2001.00490.x, PMID: 11678946

[26] Wald J Henningsson A Hanze E Hoffmann E Li H Colquhoun H . Allopregnanolone concentrations in breast Milk and plasma from healthy volunteers receiving Brexanolone injection, with population pharmacokinetic Modeling of potential relative infant dose. Clin Pharmacokinet. (2022) 61:1307–19. doi: 10.1007/s40262-022-01155-w, PMID: 35869362 PMC9439988

[27] Posner K Brown GK Stanley B Brent DA Yershova KV Oquendo MA . The Columbia–suicide severity rating scale: initial validity and internal consistency findings from three multisite studies with adolescents and adults. Am J Psychiatry. (2011) 168:1266–77. doi: 10.1176/appi.ajp.2011.10111704, PMID: 22193671 PMC3893686

[28] Kroenke K Spitzer RL Williams JBW. The PHQ-9: validity of a brief depression severity measure. J Gen Intern Med. (2001) 16:606–13. doi: 10.1046/j.1525-1497.2001.016009606.x, PMID: 11556941 PMC1495268

[29] Guy W. ECDEU assessment manual for psychopharmacology. Rockville, MD: US Department of Heath, Education, and Welfare Public Health Service Alcohol, Drug Abuse, and Mental Health Administration (1976).

[30] Newman CW Jacobson GP Spitzer JB. Development of the tinnitus handicap inventory. Arch. Otolaryngol. - Head Neck Surg. (1996) 122:143–8. doi: 10.1001/archotol.1996.01890140029007, PMID: 8630207

[31] Meikle MB Henry JA Griest SE Stewart BJ Abrams HB McArdle R . The tinnitus functional index: development of a new clinical measure for chronic, intrusive tinnitus. Ear Hear. (2012) 33:153–76. doi: 10.1097/AUD.0b013e31822f67c0, PMID: 22156949

[32] Peter N Kleinjung T Jeker R Meyer M Klaghofer R Weidt S. Tinnitus functional index: validation of the German version for Switzerland. Health Qual Life Outcomes. (2017) 15:94. doi: 10.1186/s12955-017-0669-x, PMID: 28476163 PMC5420117

[33] Adamchic I Langguth B Hauptmann C Alexander Tass P. Psychometric evaluation of visual analog scale for the assessment of chronic tinnitus. Am J Audiol. (2012) 21:215–25. doi: 10.1044/1059-0889(2012/12-0010), PMID: 22846637

[34] Henry JA Flick CL Gilbert A Ellingson RM Fausti SA. Reliability of tinnitus loudness matches under procedural variation. J Am Acad Audiol. (1999) 10:502–20. doi: 10.1055/s-0042-1748540, PMID: 10522624

[35] MedRX (2019). MedRX Tinnometer training manual

[36] Chernik DA Gillings D Laine H Hendler J Silver JM Davidson AB . Validity and reliability of the observer's: assessment of alertness/sedation scale. J Clin Psychopharmacol. (1990) 10:244–51. doi: 10.1097/00004714-199008000-00003 2286697

[37] Zeman F Koller M Figueiredo R Aazevedo A Rates M Coelho C . Tinnitus handicap inventory for evaluating treatment effects: which changes are clinically relevant? Otolaryngol Head Neck Surg. (2011) 145:282–7. doi: 10.1177/0194599811403882, PMID: 21493265

[38] Folmer RL Theodoroff SM Casiana L Shi Y Griest S Vachhani J. Repetitive transcranial magnetic stimulation treatment for chronic tinnitus: a randomized clinical trial. JAMA Otolaryngol Head Neck Surg. (2015) 141:716–22. doi: 10.1001/jamaoto.2015.1219, PMID: 26181507

[39] Sharma D Kaur S Singh J Kaur I. Role of acamprosate in sensorineural tinnitus. Indian J Pharmacol. (2012) 44:93–6. doi: 10.4103/0253-7613.91876, PMID: 22345878 PMC3271548

[40] Cohen J. A power primer. Psychol Bull. (1992) 112:155–9. doi: 10.1037/0033-2909.112.1.155, PMID: 19565683

[41] SAS Institute Inc. SAS software [computer program]. Version 9.4. Cary, NC: SAS Institute Inc. (2016).

[42] R Core Team. R: A language and environment for statistical computing R Foundation for Statistical Computing [computer program]. Version 4.2.2. Vienna, Austria: R Foundation for Statistical Computing. (2022).

[43] Wickham H. ggplot2: Elegant graphics for data analysis. In: Gentleman R Hornik K Parmigiani G editors. New York: Springer-Verlag (2016). Available online at: https://ggplot2.tidyverse.org

[44] Henry JA. “Measurement” of tinnitus. Otol Neurotol. (2016) 37:e276–85. doi: 10.1097/MAO.0000000000001070, PMID: 27518136

[45] Jufas NE Wood R. The use of benzodiazepines for tinnitus: systematic review. J Laryngol Otol. (2015) 129:S14–22. doi: 10.1017/S0022215115000808, PMID: 25858126

[46] Tunkel DE Bauer CA Sun GH Rosenfeld RM Chandrasekhar SS Cunningham ER . Clinical practice guideline: tinnitus. Otolaryngol Neck Surg. (2014) 151. doi: 10.1177/0194599814545325 25273878

[47] Jalali MM Kousha A Naghavi SE Soleimani R Banan R. The effects of alprazolam on tinnitus: a cross-over randomized clinical trial. Med Sci Monit. (2009) 15:PI55–60. 19865063

