# Supplementary figures and images for: An open-label, proof-of-mechanism trial evaluating a neuroactive steroid GABA modulator in tinnitus
Source: Front Neurol. 2025 Nov 18;16:1662226. doi: 10.3389/fneur.2025.1662226 (PMC12668930; doi:10.3389/fneur.2025.1662226)

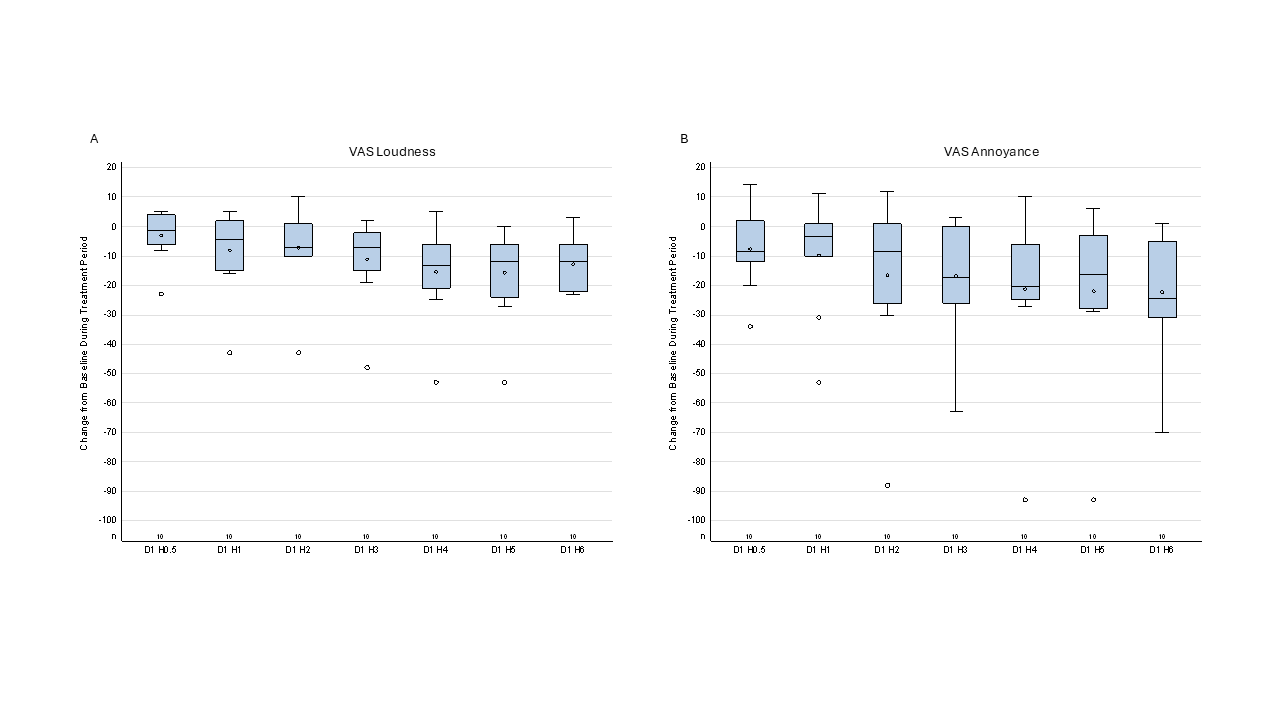

Supplement: SUPPLEMENTARY FIGURE 1 — Diagram of study selection. [file Image_1.TIF]

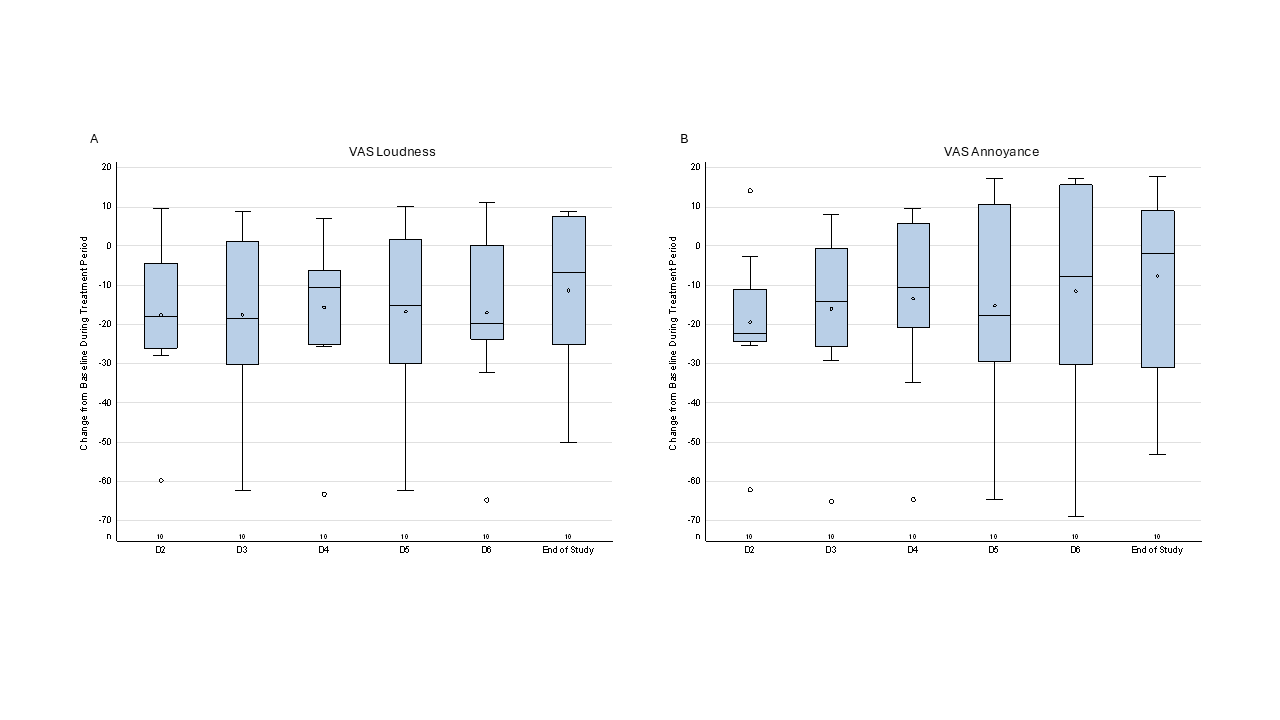

Supplement: SUPPLEMENTARY FIGURE 2 — Data from the analysis of VAS loudness (A) and annoyance (B) over the infusion period. Mean is represented by the middle open circle, median as the line, and the interquartile ranges as whiskers. [file Image_2.TIF]

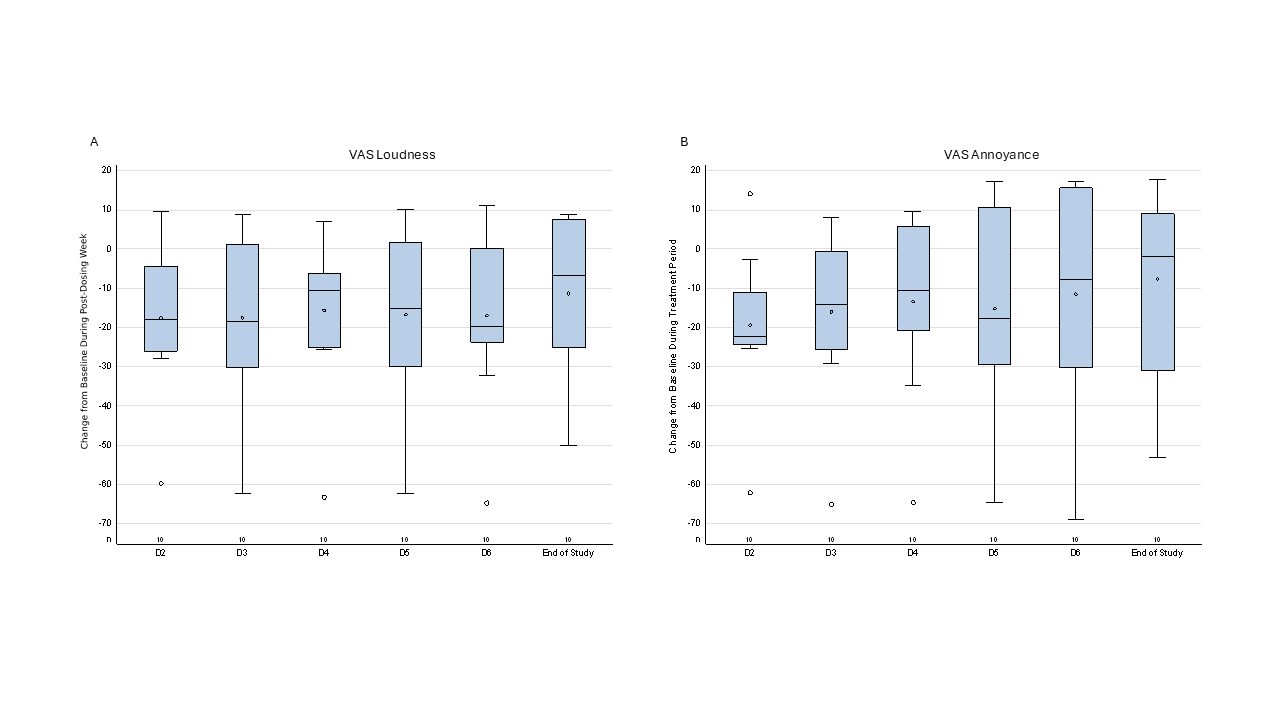

Supplement: SUPPLEMENTARY FIGURE 3 — Data from the analysis of VAS loudness (A) and annoyance (B) over the post-dosing week. Mean is represented by the middle open circle, median as the line, and the interquartile ranges as whiskers. [file Image_3.tiff]
