# Supplementary material for: An open-label, proof-of-mechanism trial evaluating a neuroactive steroid GABA modulator in tinnitus
Source: Front Neurol. 2025 Nov 18;16:1662226. doi: 10.3389/fneur.2025.1662226 (PMC12668930; doi:10.3389/fneur.2025.1662226)
Supplement: Supplementary file 4 [file Table_1.docx]

**Supplemental Table 1. Brexanolone concentrations throughout the infusion period.**

| **Timepoint (hours)** | Mean ng/mL (*SD*) |
| --- | --- |
| 0.5 | 16.3 (10.72) |
| 1 | 35.0 (13.28) |
| 2 | 63.5 (14.86) |
| 3 | 75.5 (21.22) |
| 4 | 74.1 (21.47) |
| 5 | 83.9 (24.92) |
| 6 | 67.3 (33.99) |
| 8 (after infusion) | 17.0 (6.17) |

Abbreviations: ng/mL = nanograms per millilitre; *SD* = Standard deviation.
